# Research Advances on Health Effects of Edible *Artemisia* Species and Some Sesquiterpene Lactones Constituents

**DOI:** 10.3390/foods10010065

**Published:** 2020-12-30

**Authors:** Antoaneta Trendafilova, Laila M. Moujir, Pedro M. C. Sousa, Ana M. L. Seca

**Affiliations:** 1 Institute of Organic Chemistry with Centre of Phytochemistry, Bulgarian Academy of Sciences, Acad. G. Bonchev Str., bl. 9, 1113 Sofia, Bulgaria; 2 Department of Biochemistry, Microbiology, Genetics and Cell Biology, Facultad de Farmacia, Universidad de La Laguna, 38206 La Laguna, Tenerife, Spain; lmoujir@ull.edu.es; 3 Faculty of Sciences and Technology, University of Azores, 9500-321 Ponta Delgada, Portugal; sdoffich@gmail.com; 4 cE3c—Centre for Ecology, Evolution and Environmental Changes/Azorean Biodiversity Group & Faculty of Sciences and Technology, University of Azores, Rua Mãe de Deus, 9500-321 Ponta Delgada, Portugal; 5 LAQV-REQUIMTE, University of Aveiro, 3810-193 Aveiro, Portugal

**Keywords:** *Artemisia*, clinical trials, health effects, adverse effects, anticancer, antiparasitic, artemisinin, santonin, achillin, tehranolide

## Abstract

The genus *Artemisia*, often known collectively as “wormwood”, has aroused great interest in the scientific community, pharmaceutical and food industries, generating many studies on the most varied aspects of these plants. In this review, the most recent evidence on health effects of edible *Artemisia* species and some of its constituents are presented and discussed, based on studies published until 2020, available in the Scopus, Web of Sciences and PubMed databases, related to food applications, nutritional and sesquiterpene lactones composition, and their therapeutic effects supported by in vivo and clinical studies. The analysis of more than 300 selected articles highlights the beneficial effect on health and the high clinical relevance of several *Artemisia* species besides some sesquiterpene lactones constituents and their derivatives. From an integrated perspective, as it includes therapeutic and nutritional properties, without ignoring some adverse effects described in the literature, this review shows the great potential of *Artemisia* plants and some of their constituents as dietary supplements, functional foods and as the source of new, more efficient, and safe medicines. Despite all the benefits demonstrated, some gaps need to be filled, mainly related to the use of raw *Artemisia* extracts, such as its standardization and clinical trials on adverse effects and its health care efficacy.

## 1. Introduction

*Artemisia* genus (Asteraceae family) comprise more than 2290 plant name records in the “The Plant List” database, being only 530 of these taxa with accepted Latin botanical name [1], which shows how challenging the taxonomy of this genus is. In this review, the complete accepted Latin botanical name, according to the “The Plant List” database, is presented in the first species citation, with an indication of any synonym when the latter is the one mentioned in the original publication. In the remaining citations, the genre name is abbreviated to *A.* and omitted the authority name. The *Artemisia* species are herbs and shrubs, which could be perennial, biennial and annual plants, distributed on all continents except Antarctica, mainly on Northern Hemisphere, with only 25 species on the Southern Hemisphere [2], being the Asian the zone where higher species diversity is concentrated [3,4]. They exhibit a great ability to grow on different ecosystems from the sea level to the mountains and from arid areas to wet regions, but the majority of the species live on temperate zones [2]. This ability contributed very significantly to the fact that some species of *Artemisia*, although originating in a specific zone, are now widely distributed. For example, *Artemisia vulgaris* L. is native to Europe and Asia but has now a large distribution in natural habitats worldwide, can be found in abundance in very distant areas ranging from Africa, North America to the Himalayas and Australia [5]. Some *Artemisia* species exhibit so high ability to adapt to new habitats that they become invasive species in these environments, posing a significant threat to biodiversity. This is the case of *Artemisia princeps* L. which, being native to Japan, China and Korea, is currently classified as an invasive species in Belgium and Netherlands [6] while *Artemisia verlotiorum* Lamotte is an alien invasive species in Croatia [7].

The use of *Artemisia* species in traditional medicine is well-documented [5,8,9,10,11,12,13,14,15,16,17] and demonstrates the great ethnopharmacological value of this genus. *Artemisia annua* L. and *Artemisia absinthium* L. are the best known for their uses in traditional medicine around of the World. For example, *A. annua* is cited in several ancient books as being suitable for the treatment of consumptive fever, jaundice, summer heat wounds, tuberculosis, lice, scabies, dysentery, and hemorrhoids in addition to pain relievers, while in Iran is used as antispasmodic, carminative, or sedative remedy for children [9,16,18]. In turn, *A. absinthium* has been traditionally used to treat mainly gastrointestinal diseases and as anthelmintic although for example in Italy it is also used as an antiparasitic, antihypertensive and anti-inflammatory, while in France it is also used to stimulate appetite, as an antipyretic, and emmenagogue [17,19]. However, many other species are used on each continent. For example, *Artemisia afra* Jacq. ex Willd. is one of the most widely used herbal remedies in South Africa to treat inflammation and pain [20]. It is also used to treat various ailments including coughs, colds, asthma, fever, influenza, diabetes and malaria [20], and by certain South African traditional healers to treat rhinitis [21]. *Artemisia dracunculus* L. is widely used in North America, for example by the Chippewa and Costanoan Indians as abortifacient and medicine to treat chronic dysentery, heart palpitations, wounds, colic in babies, and also to strengthen hair and make it grow [22]. In the Iranian Traditional Medicine *A. vulgaris* is used to treat cervicitis [23], while this species is reported in the ethnobotany of Karok, Kiowa, Miwok Paiute, Pomo and Tlingit areas, as a drug with several applications such as in childbirth, steam bath for pleurisy, gonorrheal sore, cold, rheumatism, headache, a ‘worm’ medicine, pains of afterbirth [24]. The traditional use of *Artemisia* species in Europe is mainly as food, spices and beverages (discussed in more detail in Section 2). However, *Artemisia* species are also used in the treatment of various diseases, as for example *Artemisia umbelliformis* Lam. and *Artemisia genipi* Weber ex Stechm. also known as Alpine wormwoods and génépis species, that are used traditionally to fight cold fever, fatigue, dyspepsia and respiratory infections, as wound-healing agents and to treat bruises, while wines aromatized with these species stimulate appetite, promote digestion, and fight the mountain sickness [10].

Even some *Artemisia* species less scientifically known such as *Artemisia ordosica* Krasch, have significant ethnomedicinal applications. This species was recorded on the traditional Mongolian and Chinese medicine books, as having a beneficial effect on the nasal bleeding, rheumatoid arthritis, headache, sore throat and carbuncle [25] and was used by Mongolian “barefoot” doctors for nasosinusitis treatment [26]. *Artemisia tripartita* (Nutt.) Rydb. was reported on Native American Ethnobotany database [27] as diaphoretic and remedy to treat cold and sore throats, while *Artemisia verlotiorum* Lamotte, distributed in all northern hemisphere, is used in Tuscany folk medicine to treat hypertension [28], and to alleviate stomach problems in Gilgit-Baltistan, Pakistan [29].

*Artemisia* species, as well as other herbal medicines, with proven pharmacological effects has been incorporated into conventional medicine. This incorporation is supported by the world health organization, which considers that traditional and complementary medicine can make a significant contribution to the goal of achieving universal health coverage by being included in the provision of essential health services [30]. Nevertheless, international research into traditional herbal medicines should be subject to the same ethical and methodological requirements as all research involving humans. Therefore, criteria to promote the safety, quality and effectiveness of the plants used in traditional medicine have been discussed and established [31,32].

Encouraged by this wide application in traditional medicine, the scientific community has dedicated itself to investigating in each *Artemisia* species evidence to support these applications. In the laboratory, the properties of the plant and extracts are tested using different models (in vitro, in vivo, clinical trial), and the active principles present in these species are wanted.

The result of this vast investigation showed the *Artemisia* species (extracts and essential oils) as exhibiting antiparasitic, anticancer and anti-inflammatory action in addition to antioxidant, wound healing, antinociceptive, immunoregulation, hepatoprotective, neuroprotective, anti-asthmatic, antidiabetic, antihypertensive, anti-adipogenic, anti-ulcerogenic, antiviral, antibacterial, antifungal, and anti-osteoporotic activities [10,11,12,13,18,19,33,34,35,36,37,38,39].

The search for bioactive compounds responsible for these biological activities has led to *Artemisia* species are privileged sources of compounds with highly diversified structures that exhibit a high level and diversity of biological activities, providing the basis for the development of new drugs, some of which are already used in clinical therapeutics [4,8,10,13,40,41,42,43,44,45,46,47].

The *Artemisia* secondary metabolites belong to the several organic compounds families [44,47,48,49] such as terpenoids [14,44], mostly monoterpenes in essential oils [31,44] and sesquiterpene lactones [40,41,50], flavonoids [14,46,51,52], lignans [52,53,54,55], alkaloids [56], steroids [14,57], phenolic acids [37,47,58] and coumarins [14,53,59], all of them well known for their large range of biological activities.

Given the large number of papers published on the theme of health effects of products related to *Artemisia* species (plant, extracts, pure compounds, studies in vitro, in vivo and clinical trials), all publications related to in vitro studies were excluded from this review. In fact, although these studies are essential for a first assessment of the species’ potential, they are the ones that are farthest from the final objective, which is, the application in patients. Thus, the results of these studies are those that weigh less in the realistic assessment of the therapeutic effects of the plant and/or its constituents.

This review is intended to gather and discuss the most impactful research concerning the health effects of edible *Artemisia* species, based on their applications as food, their nutritional composition and therapeutic applications supported by clinical studies. It is also discussed the therapeutic relevance of some sesquiterpene lactones constituents of *Artemisia* species and its derivatives.

The method consisted of searching the Scopus, Web of Science, PubMed and Google Scholar databases for original and review articles in English language, published from 2015 to 2020, while ClinicalTrials.gov was used to find registered clinical trials. For a systematic search, “Artemisia” as the primary keyword, associated with other keywords such as “chemical composition”, “nutritional”, “food”, “adverse effects” and “covid-19” for Section 2, Section 3, Section 4 and Section 7; “Artemisia” and “clinical trial” for Section 5; For Section 6 are used the name of each sesquiterpene lactone and terms like “biological activity”, “cancer”, “malaria”. The in vitro studies were excluded (NOT “in vitro”). More than 300 references were considered, and the most significant results discussed and presented here.

## 2. Use of *Artemisia* Species as Food, Spices, Condiments and Beverages

In addition to the traditional medicine applications, *Artemisia* species exhibit high food value since many of them are species used in culinary.

The most extensive use of *Artemisia* species as food is found in the countries of Europe, Asia (Japan, Korea, China and India) as well as in North America. The literature data describing the utilization of *Artemisia* species as a food, spices, condiments and beverages are summarized in Table 1.

Taxa of the *A. vulgaris* is collected and cultivated for different alimentary purposes [60,67,80]. Their leaves are one of the ingredients of kusa-mochi and hishi-mochi, two kinds of rice cakes or dumplings, one variant of which is called yomogi-mochi, yomogi being the Japanese name of these *Artemisia* species [80]. A type of soba, Japanese noodles used in soups and similar dishes, made with wheat and buckwheat also contains *A. princeps*, which gives it a green color. The young plants (leaves, stems, or shoot tips) of *A. dracunculus*, *A. dracunculoides*, *A. vulgaris*, *A. japonica*, *A. capillaris*, *A. carvifolia*, *A. indica*, *A. keiskeana*, *A. montana*, *A. schmidtiana*, *A. tilesii*, *A. tridentata*, *A. wrightii*, etc. (Table 1) can be eaten fresh in salads or cooked in soups and food supplements. *Sabzi khordan* is an Iranian (Persian) mixture of fresh herbs (served with lunch and dinner) that typically includes tarragon (*A. dracunculus*) [83]. Although the seeds of *A. dracunculoides* and *A. tridentata* are very small, they can be roasted, ground into a powder, and mixed with water or eaten raw [75]. Similarly, the seed of *A. wrightii* is crushed with water, made into balls and steamed [75].

On the other hand, the flavoring use of an *Artemisia* species is worldwide and especially of *A. dracunculus* (French tarragon, German tarragon, true tarragon or estragon) and closely related *A. dracunculoides* (Russian tarragon). The early culinary history is obscure, but the name “tarragon” is derived from *tarkhūn,* the Arabic name [83]. Tarragon became popular as a flavoring agent in the 16th century and is one of the most sought after herbs amongst gourmet chefs because of its delicate anise flavor, reminiscent of licorice. *Herbes vénitiennes* are a mixture of aromatic herbs (tarragon, chervil, parsley and sorrel) traditionally used in France to flavor butter. Tarragon vinegar is made by steeping a few fresh leafy twigs in a bottle of white wine vinegar. It is an essential ingredient of famous sauces such as béarnaise, hollandaise and tartare that classically accompany asparagus, green beans, peas and other vegetables (as tarragon cream) or chicken, meat and eggs. Leaves (preferably fresh) are used to flavor meat dishes, stews, fish dishes, salads, pickles and mustard sauces. Russian tarragon (*A. dracunculoides*) is very similar but more robust (and less aromatic). Tarragon is largely cultivated and commercialized as living plants in pots. Dried leaves of *A. argyi* are utilized as flavoring and colorant for the Chinese dish Qingtua [46], and *A. ludoviciana*—for sauces, gravies and as a garnish for pork and game [60,67]. Dried leaves of *A. vulgaris* (mugwort) make a bitter seasoning for poultry stuffing, especially for goose, and soups, and fresh leaves can be rubbed on fatty meats before roasting [61]. Some gourmands like cheese use seasoning with a mixture of wormwood (*A. absinthium*), thyme, and rosemary (at ratio 4:2:1) [84]. Small farmers in Lithuania are using a mix of wormwood and tansy (*Tanacetum vulgare*) for preservative purposes (to protect smoked meats from flies blow, other pests, and spoilage) as well as for flavoring (it gives a specific pleasant smell to the meat) [84]. The leaves of *A. frigida* are used by the Hopi Indians as a flavoring for sweet corn [68,76]. *A. abrotanum* is the sweetest *Artemisia* with slight lemon scent is a natural choice for seasoning cakes, pastries and vinegars [61]. Similarly, the leaves of *A. pallens* are delicately scented and the flowers yield a balsamic essential oil (davana) with application in baked goods, candy, chewing gum, and ice cream [61].

Many *Artemisia* species are applied in the preparation of different non-alcoholic beverages, giving them a bitter taste and alleged tonic properties. Thus, *A. absinthium*, *A. abrotanum*, *A. agryi*, *A. ludoviciana*, *A. montana*, *A. tridentata*, *A. granatensis*, etc. are consumed as herbal tea with digestive properties (Table 1). Silver wormwood (*A. arborescens*) and *A. herba-alba* are added to the green tea or the coffee in North Africa [67,78] and *A. carvifolia* has the same use in Asia [67]. Tarragon (*A. dracunculus*) is an ingredient of Georgian carbonated soft drink called *Tarkhuna* [75]. *A. maritima*, *A. abrotanum*, *A. absinthium, A. vulgaris*, etc. (Table 1) have been applied as a flavoring ingredient in beer production before the common application of hops.

Undoubtedly, the most famous *Artemisia* species employed in alcoholic drinks is *A. absinthium*, among which two are most noteworthy: vermouth and absinthe. Vermouth is a low alcoholic drink prepared from wine and a cocktail of botanical ingredients with *A. absinthium* as a principal component [85]. There are similar drinks in some countries of the Balkan Peninsula—pelin in Bulgaria [86] and vin pelin in Romania [87]. The spirit drink absinthe was created in French-speaking Switzerland in the late eighteenth century [88] and is produced by macerating *A. absinthium* leaves, anise and fennel seeds in alcohol (85 vol%) [84,89]. Wormwood (*A. absinthium*) is also used for the preparation of a bitter liqueur with lower content of alcohol (28–35 vol%) called pelinkovac (pelinkovec, pelinovec, pelen or pelin) and popular in Croatia, Serbia, Montenegro, Bosnia-Herzegovina, North Macedonia as well as in Slovenia [71].

Another popular herbal liqueurs in which *Artemisia* species present are genepy or génépi (*A. genipi* and related taxa such as *A. glacialis* and *A. umbelliformis*) [10] and ratafia (*A. abrotanum*, *A. absinthium*, *A. arborescens* and *Artemisia chamaemelifolia* Vill. [65].

## 3. Nutritional Value of *Artemisia* Species

As demonstrated above *Artemisia* species are widely consumed by human as a traditional food, a tea and dietary supplements, owing to the fact that they are rich in fatty acids, carbohydrates, dietary fiber, protein, essential amino acids, vitamins and minerals as demonstrated in Table 2.

Fatty acids (FA) have chemo-preventive effects and are pharmacologically active in chronic or degenerative diseases. Research has proved that diets rich in saturated fatty acids (SFA) are a risk factor for cardiovascular diseases unlike diets rich in monounsaturated fatty acids (MUFA) and polyunsaturated fatty acids (PUFA), which reduce or inhibit such cardiovascular disease [104]. Linoleic and linolenic acids are essential for human health growth health promotion and disease prevention [104,105,106,107,108]. They could not be synthesized endogenously in the human body and therefore they need to be supplied by food. The studies on the fatty acid profile of *Artemisia* species (Table 2) showed a very variable fatty acid content, ranging from 3.31 mg/g FW in *A. arborescens* [92] to 24.7 mg/g FW in *A. argyi* [46]. With exception of *A. jacutica* [99], *A. santolinifolia* [100] and *A. stelleriana* [92], unsaturated fatty acids (UFA) predominated in all investigated species, followed by polyunsaturated fatty acids (PUFA) and saturated fatty acids (SFA) (Table 2). Among individual compounds, linolenic and linoleic acids are the major fatty acids. These results determine *Artemisia* plants as a valuable source of unsaturated fatty acids with significance from both dietary and nutritional point of view. Palmitic acid was found to be the most abundant component in *A. stelleriana* (70%) [92], *A. princeps* (34.9%) [73] and *A. jacutica* (20.6–21.8%) [99].

Carbohydrates are important for keeping the body supply with energy and stamina. Recently, *A. sphaerocephala* carbohydrates have been the subject of a bibliographic review [109], showing their high nutritional value and versatility in terms of applications. For instance, the total amount of carbohydrates in *A. sphaerocephala* seed oil is 73% [83,110] while in *A. annua* is only 8% [93]. Recently, it has been found that the oil cake remaining after the extraction of essential oils from *A. absinthium* contains 9.4% of sugars [90]. The authors propose an application of the aqueous extracts of oil cake for the formulations of gelled desserts.

Dietary fiber possesses an ability to prevent or relieve constipation and foods containing fiber can provide other health benefits as well, such as helping to maintain a healthy weight and lowering your risk of diabetes, heart disease and some types of cancer [111]. Few *Artemisia* species have been studied for the content of dietary fiber (Table 2). The amount of crude fiber in the fresh *A. argyi* leaves, *A. annua*, *A. herba-alba* leaves and *A. sibieri* is 39.9 mg/g, 142 mg/g, 407.9 mg/g and 484 mg/g, respectively (Table 2).

Proteins play critical roles in cellular functions, structure and regulations of metabolic activities in all living organisms and have primary importance in the daily diets of consumers. Crude protein content was assessed in *A. argyi* and *A. princeps* [73], *A. herba-alba* [95,96,97], *A. campestris* [97], *A. sieberi* [101], *A. frigida* [96], *A. tridentata* ssp. *wyomingensis* [103] and *A. annua* [93]. It has been found that leaves and inflorescence of *A. annua* are rich in protein (27.1 and 18.4%, respectively) when compared to stems and roots (10.7 and 8.23%, respectively) [91]. The comparative study of the nutritional constituents of *A. princeps* and *A. argyi* showed significant difference in the content of free amino acids [73]. The content of the essential amino acids valine and phenylalanine is significantly higher in *A. argyi* (by approximately 63% and 41%, respectively) than in *A. princeps*. The amount of total essential amino acids is approximately 57% in *A. princeps* and 61% in *A. argyi*. γ-Aminobutyric acid is the main component in *A. argyi*. This acid is a natural non-protein amino acid with great therapeutic potential in neurological disorders and mental illnesses, because it acts as the major inhibitory neurotransmitter in the central nervous system [112]. Reflective of the protein content in the various tissues of *A. annua*, the highest concentration of amino acids was registered in the leaves and inflorescence and leucine was the most abundant one [91].

As can be seen in Table 2, few *Artemisia* species have been investigated for the presence of vitamins and minerals. Thus, the content of vitamin C (measured as ascorbic acid) in *A. argyi* was found to be twice higher than in *A. princeps* [73]. The content of vitamin E varies in the different plant parts of *A. annua*, from 1.19 mg/kg (stems) to 22.63 mg/kg (leaves) [91] and is significantly lower from that reported by Iqbal et al. [93]. The different values of vitamin E are probably due to the different methods used for the quantitative determination. The measured concentration of vitamin A in the different parts of *A. annua* was under detection limit level (<0.3 μg/100 g) [91]. Among minerals, potassium was detected in the highest concentration in all studied *Artemisia* species so far (Table 2) followed by calcium and magnesium (Table 2). Potassium is important in a balance for cellular metabolism and regulating transfer of nutrients to cells, maintaining blood pressure and electrolyte balance, transmitting electrochemical impulses and for the correct functioning of blood, endocrine/digestive and nervous systems, heart, kidneys, muscles and skin [113]. High levels of phosphorous were found in aerial parts of *A. frigida* [96] and *A. sieberi* [101], in *A. annua* leaves and inflorescence [91]. Phosphorous is an essential component to maintain electrolyte balance, correct functioning of brain cells, circulatory and digestive systems, eyes, liver, muscles, nerves and teeth/bones. A balance of magnesium, calcium and phosphorus is required for these minerals to be used effectively [113]. Regarding the microelements, iron, manganese and zinc in different amounts dominated all studied samples (Table 2). Probably, the functions of all these macro- and microelements in the body to maintain water balance and to stimulate normal movement in the intestinal tract can explain the traditional use of the *Artemisia* plants as an herbal tonic.

## 4. Adverse Effects Reported to *Artemisia* Species and Some Constituents

Pollen from the various *Artemisia* species is one of the most frequent and serious pollinosis causes in many parts of the world [114,115,116,117,118]. It has been verified as an allergen by nasal challenge and bronchial provocation tests, and these allergens have been shown to occur not only in its pollen but also in its leaves and stems. Studies on the immunological changes from *Artemisia* pollen allergic subjects revealed that *Artemisia* pollen can trigger not only allergic rhinitis but also asthma alone or both [118,119]. Almost half of the patients with autumnal pollen allergic rhinitis developed seasonal allergic asthma within 9 years [118]. The immunoelectrophoretic comparison of the allergen extracts from pollen of six *Artemisia* species and morphological studies on the pollen grains showed an extensive degree of similarity and cross-reactivity between the studied species [120]. Screening of both Korean and Norwegian patient sera against extracts from *A. vulgaris* and *A. princeps* showed that both groups of patients had the same pattern of reactivity towards both extracts [120]. The mugwort (*A. vulgaris*) pollen contained allergenic substances with IgE reactivity [121], which can cause immediate Type I allergic reactions such as anaphylactic shock [122]. Another study on the pollen collected from plants across Europe has shown that the highest levels of endotoxin were detected on *A. vulgaris* pollen [123]. The investigation on *Artemisia* pollen allergenicity revealed significant daily, seasonal and species-specific variability [124]. The analysis of *Artemisia* pollen concentrations evidenced the presence of a bimodal curve with two peaks. The first peak was attributed to *A. vulgaris* (early flowering species) and the second one—to late flowering species *(A. campestris*, *A. annua*, *A. verlotiorum*, etc.) [115,124]. The authors supposed that the spread of these species could affect human health, increasing the length and severity of allergenic pollen exposure in autumn.

Skin contact with some members of the genus *Artemisia* can cause dermatitis or other allergic reactions in some people [125,126,127,128]. Several cases of contact dermatitis are described in the literature [129,130,131,132]. Mugwort (*A. vulgaris*) has demonstrated a medium sensitizing capacity in guinea pigs [133]. According to Park [134], nearly 43% of patients with allergic rhinitis and asthma have positive reactions to mugwort on skin prick testing. Wormwood tea (*A. absinthium*) induced positive patch test reactions in 13 of 19 Compositae-allergic patients [135]. Erythema multiforme, is probably an expression of a delayed hypersensitivity reaction and appears clinically as acute or chronic dermatitis of exposed sites [127,128]. Skin contact dermatitis caused by *Artemisia* species is attributed to the presence of sesquiterpene lactones [129,136,137].

Absinthe and the use of wormwood extracts (*A. absinthium*) for food purposes were prohibited around the years 1910–1920 in many countries as their consumption was associated with a range of severe adverse symptoms called absinthism, including convulsions, blindness, hallucinations and mental deterioration [89,138,139,140]. Padosch et al. [139] reviewed the available data concerning medical and toxicological aspects experienced and discovered before the prohibition of absinthe. Numerous studies did not give a clear answer whether the toxicity is due to thujone alone, to a combination of the alcohol and thujone or whether it can be traced back to toxic components used in the manufacture of absinthe liqueur [89,140]. Nowadays, *A. absinthium* is permitted in foods and alcoholic beverages according to the regulation of the European Parliament and Council [141]. In addition, European Food Safety Authority (EFSA) states that thujone content in alcoholic beverages, including absinthe, must not exceed 10 mg/kg [142], while the European Medicines Agency (EMA) also proposed a daily maximum intake of thujone in *Absinthii herba*, which was set at 3.0 mg thujone/day/person as acceptable for a maximum duration of 2 weeks in the wormwood monograph [143].

Some *Artemisia* species such as *A. vulgaris* [144], *A. herba-alba* [145], *A. annua* [146,147], *A. arborescens* and *A. douglasiana* Besser ex Besser [67] are used in regulating fertility and should be avoided in pregnancy. Thus, the consumption of *A. herba-alba* during pregnancy of mice offspring significantly decreased the fertility ratio and increased the weight and body size of preweaning offspring mice [148]. In addition, administration of *A. herba-alba* prolonged the time of completing the reflex response of surface righting, negative geotaxis, cliff avoidance and jumping test of mice offspring. In another study, treatment of pregnant rats with *A. kopetdaghensis* “Krasch, Popov & Lincz. ex Poljakov” hydroalcoholic extract (200 and 400 mg/kg) from the 2nd to 8th day of pregnancy led to 30 and 44% abortion in animals but had no significant effect on duration of pregnancy, average number of neonates, and weight of neonates [149]. The abortifacient effect of *A. kopetdaghensis* was attributed to the high content of camphor, for which is known that can crosses the placenta [150]. Gomes et al. [151] reviewed the results of non-clinical and clinical studies with artemisinin derivatives, their mechanisms of embryotoxicity and discussed the safety of their use during pregnancy. The mechanisms of embryotoxicity are not completely understood, but might not be so relevant for humans, considering the short time of treatment (3–7 days) compared with the longer period of target cell formation in the human embryo (~3 weeks).

The sesquiterpene lactone artemisinin isolated from the herb *A. annua* and its derivatives (artemether, arteether, and sodium artesunate) are successively applied in the malaria chemotherapy [152]. Extensive studies and meta-analyses of thousands of patients did not show serious side effects [152,153,154,155,156], although proper monitoring of adverse side effects in developing countries might not be a trivial task [155]. Common side effects were nausea, vomiting, and diarrhea, which are also symptoms of malaria itself. Efferth and Kaina [157] have summarized the available data on toxicity studies (neurotoxicity, embryotoxicity, genotoxicity, hemato- and immunotoxicity, cardiotoxicity, nephrotoxicity, and allergic reactions) in cell culture, animals (mice, rats, rabbits, dogs, monkeys) and in human clinical trials of artemisinin and its derivatives and concluded that artemisinin did not cause toxicity if are taken in appropriate doses for short periods.

## 5. Therapeutic Uses of *Artemisia* Species Based on Clinical Trials

Encouraged by long traditional use of many *Artemisia* species for treatment of various ailments, research into their pharmacological effects has been carried out and seem to support the traditional applications [5,12,15,16,17]. In this regard, *Artemisia* species and their biologically active compounds have already been introduced as antimalarial, antioxidant, cytotoxic, antispasmodic, anthelmintic, antinociceptive, neuroprotective, anti-inflammatory, and antimicrobial agents, among others [16,44]. It is noteworthy that although *Artemisia* species have been intensively studied in vitro as cytotoxic agents, there are no reports on their clinical evaluation for cancer therapy in humans [4]. However, one report by Saeed et al. showed that supplementing pet food with an *A. annua* formulation (Luparte^®^) clearly improved survival prognosis in veterinary treatment of small tumors [158].

Nevertheless, clinical evaluations of *Artemisia* species for a range of other diseases have been carried out [4]. The effect of *A. annua* in traditional medicine in China for treating fever, inflammation and malaria [9] have been evaluated in clinical trials for stiffness and functional limitation associated with osteoarthritis of the hip and knee, pain management, experimental heterophyid infection and treatment of malaria [159,160,161].

*Artemisia dracunculus* has been used for glycemic control, insulin sensitivity, and insulin secretion [162] and likewise, *A. princeps* was evaluated for the same effects in subjects with impaired fasting glucose and mild-type 2 diabetes [163] and *A. absinthium* in the control of diabetes type 2 [164]. 

Ointments and liniments of *A. absinthium* can be effective in the treatment of knee osteoarthritis [165]. Based on the suppressor activity of *A. absinthium* compounds on tumor necrosis factor alpha (TNF-α) and other interleukins [166], Krebs et al. [167] established the curative effect of this *Artemisia* species in patients with Crohn’s disease. There was improvement in symptoms after treatment with dried powder of the plant together with a conventional therapy, and a cardamonin present in the plant was considered responsible for the anti-inflammatory activity.

In addition to being widely used clinically to treat itching in icteric and dialytic patients, owing to its anti-histaminic and anti-allergenic effects, *A. vulgaris* (mugwort) lotion has also provided good results in patients with post-burn hypertrophic scars [168]. 

Recently, the preventive effect on hepatitis B cirrhosis of *A. capillaris* decoction combined with the entecavir has been evaluate by a randomized, double-blind and placebo controlled clinical trial (Chinese Clinical Trial Registry: ChiCTR1900021521), to assess its efficacy and safety [169].

*Artemisia annua* and *A. vulgaris* are the species of the genus that produce the highest levels of allergens in their pollen, being one of the main causes of seasonal allergic rhinitis (“hayfever”). Lou et al. [170] carried out a phase III clinical trial (ClinicalTrials.gov identifier: NCT03990272) from March 2017 (approximately 4 months before the local natural *Artemisia* pollen season) to October 2017, involving patients from 13 centres across Northern China. The aim was to test the efficacy and safety of sublingual immunotherapy (SLI) with drops of *A. annua* for allergic rhinitis related to this plant. Results indicated that *A. annua* was a safe and significantly effective therapy. However, longer term follow-up is required, particularly to determine the mechanism of action.

Based in previous study where Xiao et al. [25] demonstrated using in vivo models, the ability of *A. ordosica* Krasch. extracts to control the allergic inflammatory response in rhinitis, clinical trials using nasal spray preparations of *A. abrotanum* containing its essential oils and flavonols have been performed with good results [171].

Munyangi et al. [172] published a randomized controlled clinical trial reporting far superior cure rates of *A. afra* and *A. annua* infusions than with artemisinin combination therapy (artesunate—amodiaquine), in the treatment of malaria. Contrastingly, a recent review by Toit and van der Kooy [15] concluded that tea infusions do not have in vitro activity, and in fact contain no artemisinin. Another randomized large-scale double-blind controlled trial on *A. annua* and *A. afra* tea vs. praziquantel for the treatment of schistosomiasis was documented by Munyangi et al. [173]. Controversially, Gillibert found scientific and ethical issues such as the article on schistosomiasis referring to the same ethics committee registration number as the malaria article [174].

Sensitive skin was initially believed to be an unusual reaction occurring in only a small subset of individuals. However, during recent decades, it has been shown to affect half the population of the world [175]. Accordingly, extensive in vitro, preclinical, and clinical research with artemisinin and its derivatives has been undertaken, notably into their anti-inflammatory, immunomodulatory and antioxidant properties [176]. Yu et al. [177] tested the effectiveness of cosmetics containing *A. annua* extract in repairing sensitive skin. In this study, the xylene-induced ear swelling and human clinical efficacy tests were used, and the authors found that applications containing *A. annua* extract can inhibit inflammation, repair the skin barrier, improve damaged skin, and reduce redness and other sensitive skin symptoms. Aside from this, its leaves are eaten in salads in some Asian countries and in the United States, and several companies currently sell ground leaves and their extracts as dietary supplements [178].

## 6. Some Sesquiterpene Lactones Constituents of *Artemisia* Species with High Clinical Relevance

The pharmaceutical industry has always been interested in the secondary metabolites produced by plants, for the treatment of diseases, in cosmetics, dyes, fragrances and flavorings [179]. The *Artemisia* species are well known by its content of sesquiterpene lactones [40,41,43,50]. These family of compounds have been studied and reveal high therapeutic potential [180,181]. Here are presented some of the most studied and promise sesquiterpene lactones constituents of edible *Artemisia* species (does not intent to be an exhaustive list) which, due to its medicinal properties discussed above, could contributes to the benefits effects of the *Artemisia* species. Sesquiterpene lactones such as arglabin parthenolide, cynaropicrin, helenalin, costunolide and thapsigargin identified in species of the genus *Artemisia* [40,41,50,180] and other genera, exhibit high pharmacological potential, including in in vivo studies and clinical trials, as demonstrated and discussed very recently [181]. So, they will not be considered in this work. The most recent and relevant experimental evidence of other sesquiterpene lactones medical potential will be highlighted discussed below. In this selection, was considerate mainly the in vivo and clinical studies, once they are the last steps of new drugs development and their results are the most significant to drug development.

**Figure 1:**
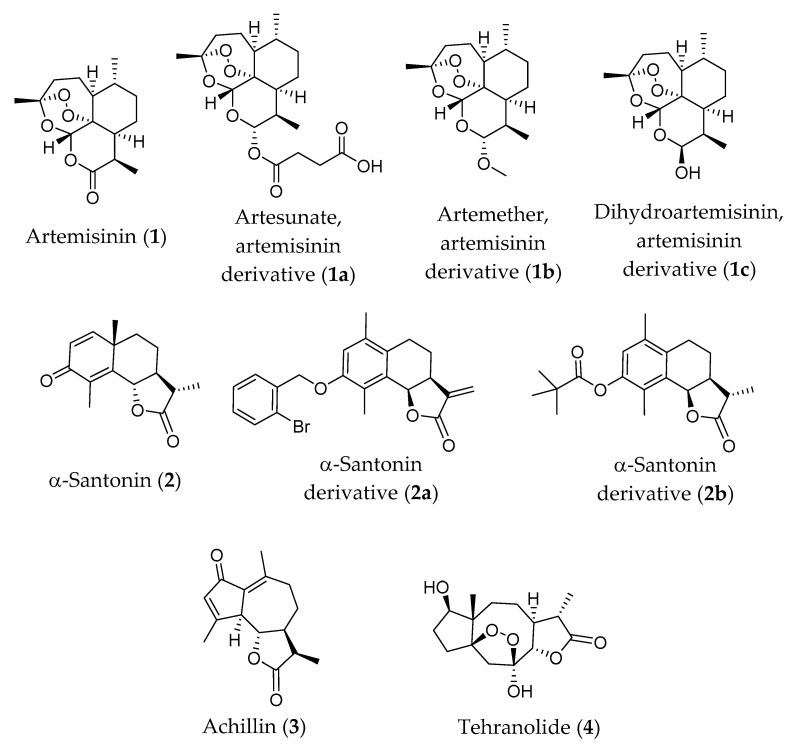
Chemical structures of some sesquiterpene lactones constituents of edible *Artemisia* sp. and derivatives with pharmacological relevance.

The chemical structures of the selected sesquiterpene lactones discussed below are indicated in the Figure 1.

### 6.1. Artemisinin and Its Derivatives

Artemisinin (**1**) is a sesquiterpene lactone with an unusual peroxide bridge (cadinene sub-group), that was discovered and isolated from the Chinese herb *A. annua* by Tu YouYou in the early 1970s [182], who was awarded the Nobel Prize in 2015, for discoveries concerning the novel artemisinin therapy against malaria.

Artemisinin (**1**) has low solubility in both water and oil, resulting in weak erratic absorption after oral administration. It also has a short half-life and high first-passage metabolism [183]. Therefore, preserving its pharmacophore, a series of derivatives were designed. The 5–10 times more potent hemiacetal, dihydroartemisinin (DHA, **1c**), also known as dihydroqinghaosu or artenimol, is produced by reducing the lactone. Alkylation of the hemiacetal yields arteether (artemotil) and artemether, while artesunate is reached by acylation of the hemiacetal with succinic acid [184]. In in vivo systems, artesunate (**1a**) and artemether (**1b**) are converted back to dihydroartemisinin (**1c**). The most clinical widely used derivative is artesunate [185]. These derivatives showed better efficacy, tolerability, and oral bioavailability (all well absorbed by mouth and rapidly eliminated) than artemisinin, as well as minimal adverse effects [186,187]. Artemisinin (**1**) and its derivatives have been highlighted for their potent activity against species of *Plasmodium* genus responsible for malaria, as well as in the treatment of leishmaniasis, schistosomiasis and trypanomiasis [188,189,190]. They have also shown efficacy on several cancer lines, along with anti-inflammatory activity, modulating the immune response by regulating cell proliferation and cytokine release [191,192,193]. They also have anti-ulcerous [194], antinociceptive [195], antiviral [196], antifungal [180], and antibacterial [197] activities, besides being effective in other disorders such as respiratory [198], and related to metabolic syndromes, including obesity, diabetes and atherosclerosis [199,200], being many of the effects mentioned evaluated in in vivo models

#### 6.1.1. Antiparasitic Activity In Vivo and Clinical Trials

Malaria is the most common tropical disease and is caused by protozoan parasites of the genus *Plasmodium* (*P. malariae*, quartan fevers; *P. vivax* and *P. ovale*, tertian fevers; *P. falciparum*, malignant tertians and *P. knowlesi*) [201]. Artemisinin (**1**) and its derivatives contain a 1,2,4-trioxane moiety responsible for the drug’s action mechanism [193,202], which despite intensive study remains debatable. The compound is believed to act in two phases. Firstly, the haem iron attacks and breaks its endoperoxide linkage to produce oxy and carbon free radicals. In the second step, the latter free radical alkylates specific malarial proteins, killing the parasite. artemisinin (**1**) also has another target, the mitochondria and endoplasmic reticulum, where it inhibits PfATP6, the enzyme responsible for Ca^++^ delivery into vesicles. This is a parasite-encoded sarcoplasmic-endoplasmic reticulum calcium ATPase (SERCA), which is crucial for parasite development [193,201,202,203,204,205]. Furthermore, compound **1** exhibits a direct antiparasitic effect against several *Leishmania* species, by increasing NO production and iNOS expression in uninfected macrophages, and an indirect immunomodulatory effect. In addition, treatment with artemisinin (**1**) in a BALB/c mouse model led to significant reduction in splenic weight, a strong inhibition of parasites, and a restoration of Th1 cytokines such as interferon-γ and interleukin-2 (IL-2) [206].

The 11th World Malaria Report 2018 by the WHO estimated a 2 million increase in global malaria cases with respect to 2017. Examples of growing challenges are urban malaria, the parasite’s drug resistance, malaria in pregnancy, resistance towards insecticides, etc. [207]. Artemisinin (**1**) and its analogues **1a**, **1b** and **1c** show marked activity against multidrug resistant strains of *Plasmodium* species and cerebral malaria both in vivo and in vitro. For this reason, the WHO recommends them as first choice treatment as part of artemisinin combination therapy (ACT) [208].

Artesunate (**1a**) is widely used to treat multidrug-resistant malaria [209,210]. The African Quinine Artesunate Malaria Trial multicenter study (AQUAMAT) conducted clinical trials with it in more than 5400 children under 15 years of age with severe malaria. As a result of these, the WHO revised the guidelines for malaria and recommended intravenous artesunate as a choice to treat severe malaria [211,212].

After a cluster-randomized trial carried out in several African countries, it was concluded that rectal derivative **1a** takes 4–6 h to reduce parasitemia and affect progression of the disease. Consequently, when malaria patients cannot be treated orally, rectal artesunate prior to hospital referral can prevent death and disability. In fact, the WHO recommends this, to reduce the risk of death or permanent disability [213].

In 2019, as part of a double-blind, randomized, placebo-controlled trial in Mozambique by Dobaño et al. [214], monthly chemoprophylaxis with sulfadoxine-pyrimethamine plus artesunate (**1a**) (ASSP) was tested to selectively control timing of malaria exposure during infancy. It was observed that a balanced proinflammatory and regulatory cytokine signature (probably by innate cells), around 2 years of age, is associated with a lower risk of clinical malaria. In addition, excellent results were obtained with ASSP therapy in patients affected with uncomplicated *P. falciparum* in India, obtaining an 84.1% cure rate. The 15% failure was due to an artemisinin-resistant isolate [215].

Recent reports highlight the capacity of iron oxide nanoparticles to enhance the efficacy of artesunate (**1a**). In fact, this combination was efficient to retard growth of *P. falciparum* at a reduced drug concentration, with significant damage to macromolecules mediated by enhanced ROS production. Its efficacy against the artemisinin-resistant strain of *P. falciparum* is noteworthy, which suggests artesunate can be developed into a potent therapeutic agent against multidrug-resistant strains [216]. Despite the success of artemisinin derivative artesunate (**1a**), the two major antimalarial policy options are dihydroartemisinin (**1c**)–piperaquine (DHA–PQP) and artemether (**1b**)–lumefantrine (AL) “first-line treatment of uncomplicated *P. falciparum* malaria worldwide” [217]. The benefits of DHA–PQP in children have been validated in endemic countries [218]. The AL combined therapy exerts its effects against the erythrocytic stages of *Plasmodium* spp. In the body, artemether (**1b**) is rapidly metabolized into the active metabolite dihydroartemisinin (**1c**). It is thought that **1b** derivative provides rapid symptomatic relief by reducing the number of malarial parasites. However, lumefantrine has a much longer half-life and is believed to clear residual parasites. Extensive clinical trials of the combination were carried out against *P. falciparum* in China and elsewhere, the success of which led to marketing the combination worldwide under the name “Coartem”. This resulted in many independent comparative drug studies, which further confirmed its efficacy [219,220].

A prospective, open label, non-randomized, interventional clinical trial of artemether (**1b**)–lumefantrine (AL) combined therapy was conducted in Zambia. It involved 152 HIV-infected patients with uncomplicated falciparum malaria, who were on efavirenz-based anti-retro-viral therapy. They received a 3-day directly observed standard AL treatment and were followed up until day 63. The results showed that while AL was well tolerated and efficacious in treating uncomplicated falciparum malaria, 16.4% of the participants had a recurrent malaria episode by day 42. This highlights the need in this sub-population for additional malaria prevention measures after treatment [221].

Furthermore, a systematic review compared the efficacies of artemether (**1b**)–lumefantrine (AL) and dihydroartemisinin (**1c**)–piperaquine (DHA–PQP) with or without primaquine (PQ) on the risk of *P. vivax* recurrence [222]. It revealed that administration of DHA–PQP considerably reduced *P. vivax* recurrence by day 42, compared with AL, although at day 63 the risk of recurrence following DHA–PQP was also reduced substantially by co-administration of PQ.

Malaria causes serious maternal and fetal complications, for this reason control of infection in in pregnancy is very important. Centers for Disease Control and Prevention (CDC) recommended artemether (**1b**)–lumefantrine combined therapy as an additional option for treatment of pregnant women with uncomplicated malaria in the United States during the second and third trimesters of pregnancy, at the same doses recommended for non-pregnant adults [223,224].

#### 6.1.2. Antitumor Activity In Vivo and Clinical Trials

Artemisinin (**1**) and its derivatives (**1a**, **1b** and **1c**) are very potent anticancer compounds, highly selective on cancer cells with almost no side effects on normal cells. This specificity is due to certain tumor cell characteristics, such as increased metabolism and the high concentration of ferrous ion required to assist their rapid proliferation. There is indeed a high concentration of transferrin, an iron transporter protein situated on the surface cell, and also a susceptibility to reactive oxygen species (ROS) [225]. Furthermore, interest in artemisinin (**1**) and its derivatives resides in their minimal toxicity and adverse effects, which suggest the possibility of utilizing them as antineoplastic drugs [186].

Recent studies have shown that compound **1** and derivatives inhibit the growth of numerous types of neoplasm cells, including breast, ovarian, prostate, lung, colon, leukemia, pancreas, melanoma, renal, hepatic, gastric, and CNS cancer cells. Several reviews have been published in the last few years describing the outstanding antitumor activities of artemisinin (**1**) and derivatives upon different pathways in human cancer cells [186,198,226,227]. Therefore, we will only point to the most relevant aspects of the in vivo and clinical trials carried out.

The antitumor mechanism of artemisinin (**1**) is also based on cleavage of its endoperoxide bridge by the ferrous iron in cancer cells and formation of ROS. Such free radicals produce cell alterations such as apoptosis, DNA damage, autophagy and cell cycle arrest G0/G1 [227]. They can also inhibit angiogenesis by inhibiting the secretion of VEGF, VEGFR2, and KDR/flk-1 in tumors [228,229]. They also may affect signaling pathways and transcription factors associated with tumor growth, including the Wnt/β-catenin and AMPK pathways, nitric oxide signaling, NF-κB, CREBP, MYC/MAX, mTOR, and AP-1 [230]. 

Some derivatives have reached the phase of clinical trials against several cancers such as breast, cervical, hepatocellular carcinoma, non-small cell lung, squamous cell laryngeal [229,231,232]. However, insufficient large-scale clinical studies have been conducted on their applications in cancer therapy.

Artenusate (**1a**) was used in phase I clinical trial to treat metastatic breast cancer (ClinicalTrials.gov identifier: NCT00764036) and concluded that 200 mg per day are recommended for future trials [233]. This compound (**1a**) is also being tested regarding colorectal cancer phase I (ISRCTN registry: ISRCTN05203252) and its safety and efficiency in stage II/III to the same disease (ClinicalTrials.gov identifier: NCT02633098), as treatment in patients with cervical dysplasia (ClinicalTrials.gov identifier: NCT02354534) and as treatment of HPV-associated anal intraepithelial neoplasia (ClinicalTrials.gov identifier: NCT03100045). Recently, Trimble et al. [234] assess for the first time the safety and efficacy of intravaginal artesunate (**1a**) to treat cervical intraepithelial neoplasia 2/3 (CIN2/3) with good results which support continuing phase II clinical (ClinicalTrials.gov identifier: NCT04098744). A phase-one study was also conducted to evaluate its safety and pharmacokinetic properties when administered orally in patients with advanced hepatocellular carcinoma (ClinicalTrials.gov identifier: NCT02304289) [235]. One double-blind placebo-controlled trial consisted of giving human colorectal cancer patients oral compound **1a** prior to surgery. During a median follow-up of 42 months, 1 patient in the artesunate group had a recurrence of colon cancer compared to 6 patients in the placebo group [236].

Artemether (**1b**) has also been included in a phase I/IIa study to assess its potential use in treating subjects with advanced solid tumors (ClinicalTrials.gov identifier: NCT02263950) [235]. Another report describes beneficial improvement in a patient with pituitary macroadenoma treated with it for 12 months [237].

The pilot clinical phase I/II trial of dihydroartemisinin (**1c**) [238] against advanced cervical carcinoma indicates that after three weeks of treatment in ten women, the majority showed improvement in the signs and symptoms. This included the vaginal discharge and pain, with no evidence of severe toxicity. These patients had a lower expression of epidermal growth factor receptor (EGFR) and Ki-67 oncogenes.

Furthermore, with the goal of increasing the antineoplastic effect of these drugs, the combination of the usual chemotherapy with artemisinin or its derivatives has been investigated, showing that their multifactorial action on various pathways may improve overall activity [232]. Interestingly, Wang et al. [239], demonstrated that dihydroartemisinin (**1c**) improves the anticancer effect of gemcitabine, a drug used in pancreatic cancer, which develops resistance over time. They confirmed by in vitro and in vivo analysis that compound **1c** induced increased growth inhibition and apoptosis 4- and 2-fold, using both drugs and alone, respectively.

Tilaoui et al. [240] observed a synergistic effect when they used vincristine and artemisinin (**1**), in combination, against murine mastocytoma (P815) cells. A randomized controlled trial with artesunate (**1a**) combine with a chemotherapy regime using vinorelbine plus cisplatin, in patients with advanced non-small cell lung cancer (NSCLL) shown that this treatment can raise the short-term survival rate and prolong the progression time, without extra side effects [241]. Liu et al. [242] found that a combination of artesunate (**1a**) with lenalinomide, commonly used for the treatment of multiple myeloma, caused an impressive enhancement of antineoplastic activity in polyploid cell lines [243], while Singh and Verna [244] described significant improvement and 70% reduction in tumor size after 2 weeks of treating a patient diagnosed with stage II cancer of the larynx with the same compound **1a** (50 mg). Additionally, the first long-term treatment of two patients with metastatic uveal melanoma with artesunate (**1a**), in combination with standard chemotherapy, was reported [245]. The standard therapy alone was ineffective in stopping tumor growth, while the disease was stabilized after adding compound **1a** to this chemotherapy, followed by objective regressions of spleen and lung metastases [245].

Another characteristic of tumors and cancer cells is their ability to develop resistance to chemotherapy due to their rapid cell-division rate and genetic mutations [246]. In this context, Reungpatthanaphong et al. [247] report that artemisinin (**1**), artesunate, and dihydroartemisinin (**1c**), combined with doxorubicin and pirarubicin, increased the cytotoxic effect induced by pirarubicin or doxorubicin only in MDR cell lines. They proposed that artemisinin and its derivatives reverse the MDR phenomenon at the mitochondrial level.

Currently, another series of derivatives are being explored that present a longer plasma life and are more powerful and effective at lower concentrations. These include artemisinin dimers and trimers, hybrid compounds, and tagging of the compounds to molecules that are involved in the intracellular iron-delivery mechanism. These compounds are promising potent anticancer agents that produce significantly less side effects than conventional chemotherapeutic agents [226].

### 6.2. α-Santonin and Its Derivatives

Santonin (C_15_H_18_O_3)_ (**2**) (Figure 1) is an eudesmanolide sesquiterpene lactone first isolated from A. santonicum by Kahler in 1830 [248], but it is much less known than artemisinin (**1**). Santonin exists in two isomeric forms, α-santonin and β-santonin [249], with α-santonin being the most studied form due to its higher stability [250]. 

Its isolation and characterization proved an arduous and grueling task for chemists at the time [251]. Its mevalonoid biosynthesis pathways has long been studied, revealing that it is formed by a methylene reduction, C-1 hydroxylation and C-3 oxidation of the precursor costunolide, sharing an identical initial pathway [252,253]. The synthesis of santonin (**2**) was first described by Marshall and Wuts in 1978 [250] and involved the reduction-alkylation of m-toluic acid with lithium in ammonia.

Santonin is the most abundant sesquiterpene lactone in *Artemisia* cina Berg ex Poljakov and was isolated from several *Artemisia* species [254,255,256], including some edible species such as *A. absinthium*, *A. frigida*, *A. tridentata* [34,255]. *Artemisia* santonicum is commonly referred to as “wormseed”, a name historically attributed to the plants’ anthelmintic (“worms”) activity [251]. Indeed, santonin was one of the most frequently used treatments for intestinal nematode infections up until the 1970s [257]. Nowadays santonin has fallen out of use due to a wealth of better anthelmintic therapeutics [258]. Nevertheless, santonin has been revealed to have many other bioactivities worthy of investigating. In a recent publication [259] santonin proved to have potent insect growth inhibitory effect on the cotton bollworm, *Helicoverpa armigera*, a widespread pest of significant agricultural and economic impact. In this work, the researchers showed that a 2 mg/mL dose of santonin in wet feed would cause a significant (~80%) decrease in larval weight compared to the control. This is explained by the results obtained from the excised midguts, which revealed that trehalase activity had decreased to 32% of the control in treated larva. Trehalase is an important enzyme in the metabolism of trehalose, an abundant simple sugar in plants. This enzyme inhibitory activity could also be allied with santonin’s effect of rupturing insect midgut cell lining [260]. Overall, this shows there is clear signs of a potential value for the compound in possible environmentally friendly pesticidal formulations, as well as a continued interest in the compound. We would also like to note the high quality of this publication, particularly in its clarity of language and presentation of data, something which is sorely missed in similar publications.

In modern times attention has mostly been devoted to α-santonin derivatives as opposed to the actual compound. This is because α-santonin’s chemical structure lends itself very well to modifications, the compound a cheap and easy to use platform for drug synthesis [261]. Indeed, two reviews were recently published [256,261] about α-santonin derivatives and how different structural changes affect their bioactivities. In vitro results are much more abundant for santonin derivatives, mainly detailing the synthesis of novel cancer therapeutic agents. Santonin tumor inhibitory derivatives have included a diacetoxy acetal form [262]; spiro-isoxazoline and spiro-isoxazolidine derivatives [263,264]; cinnamic acid derivatives [265], immunosuppressants [266]; anti-inflammatory bromoketone [267,268,269]. Given the scope of the previously cited reviews, here it will be highlighting the main recently published derivatives showing potent in vivo effects.

Regarding in vivo effects, a recently published work [270] presents an α-santonin derivative (a benzyl ether derivative containing *o*-bromine named (3a*S*,9b*R*)-8-((2-bromobenzyl)oxy)-6,9-dimethyl-3-methylene3a,4,5,9b-tetrahydronaphtho[1,2-b]furan-2(3*H*)-one) (compound **2a**, Figure 1) with potent anti-inflammatory bioactivity. In this work [270], the α-santonin derivative **2a** was synthesized and screened for in vitro activity where it was the most potent one and selected for further in vivo assays. This compound was administered orally to an arthritis rat model with very similar characteristics to rheumatoid arthritis human patients. Rats were treated with doses of 5 and 20 mg/kg (*w*/*w*) per day of the selected derivative **2a** and monitored for arthritic disease progression. Results showed a significant improvement of arthritis symptomatology in the treated rats, with ~40% lower clinical disease scores (associated with the degree of limb swelling) and significant reduction of hind paw volume. It is also worth noting that the response seems to not be dose-dependent, since both doses tested revealed almost identical activity. After mechanistic studies, the authors attribute this effect to the selective bonding of the derivative with the active site of UbcH5c, a key enzyme for ubiquitination during TNF-α-triggered activation of the NF-κB inflammatory pathway. These results seem to indicate the potent anti-inflammatory bioactivity of this novel derivative **2a**, with potential future applications in the development of a new anti-rheumatoid arthritis (RA) drug.

However, some criticism can be levelled at the work of Chen et al. [270], primarily for the way the researchers measure disease progression; their score system seems simplistic and overly reliant on somewhat subjective and qualitative observation (swelling vs. no swelling), rather than more quantitative records and measures, rendering it quite unwieldy and difficult to understand. Another addition to this work we consider beneficial would have been to include a positive control. By including a known and widely used anti-rheumatic in this in vivo assay, we could assess the bioactivity of this novel compound by comparison with a known therapeutic. We believe more studies are required in order to better understand this new agent, and hope to see clinical trials in the near future.

Novel immunosuppressant drugs are currently in very high demand due to the high cost and serious side effects of currently used therapeutics [271]. A very interesting research was published in 2017 [272], exhibiting a novel α-santonin derivative with in vivo immunosuppressant properties. In this paper [272] the authors describe the synthesis of several O-aryl/aliphatic ether, ester and amide α-santonin derivatives and the in vitro assay for immunosuppressant bioactivity, showed one particular compound exhibiting ~75–80% proliferation suppression rates for B and T lymphocytes. This derivative, a trimethyl acetate ester α-santonin analogue (compound **2b**, Figure 1), was used for in vivo immunosuppression testing using BALB/c mice. Rats were injected with 6.25 mg/kg (*w*/*w*), 12.5 mg/kg (*w*/*w*) and 25 mg/kg (*w*/*w*). Rat humoral immune response was assessed by quantification of post-challenge antibody production and cell mediated immune response was assayed by post-challenge left hind footpad thickness measurement. Results were impressive: humoral response was suppressed by 28% with the lowest dose (6.25 mg/kg (*w*/*w*)), and 41% at the highest dose (25 mg/kg (*w*/*w*)); and cell mediated immune response was suppressed by ~30% in the medium and highest doses. These results were comparable to the positive control cyclophosphamide regarding humoral response but fell considerably shorter of the positive control in the cell mediated response assay. Nevertheless, these results show that the novel derivative synthesized is capable of both humoral and cell mediated immune suppression, with reasonable potency, making it a prime candidate for future drug development. More tests with this compound, particularly exploring the mode of action, which was not presented by Dangroo et al. [272] and future clinical trials are expected.

From these studies and cited reviews, it is concluded that santonin and its derivatives are highly interesting, with new papers and bioactivities being constantly researched and published. It is expected to see some of these compounds enter pre-clinical testing soon.

### 6.3. Achillin

Achillin (**3**) (Figure 1) a sesquiterpene lactone (C_15_H_18_O_3_) of the guaianolide class, first identified in *Achillea millefolium* L. (syn *Achillea lanulosa* Nutt.) in 1963 by White and Winter [273] and synthesized in 1967 [274].

Although achillin *(***3**, Figure 1*)* is mainly extracted from plants of the *Achillea* genus, it has also been identified in edible *Artemisia* species such as *A. capillaris* [275], *A. frigida* [276], *Artemisia feddei* H. Lév. & Vaniot [277] and *A. ludoviciana* [278]. It has also been identified in plants of other species, such as *Taraxacum platycarpum* [279] and *Anthemis scrobicularis* [280]. Achillin biosynthesis pathway starts with the eudesmane skeleton of α-santonin, and involves the hydrolysis of an acetate precursor, followed by epimerization at C-11 and finishing with an allylic oxidation [281].

The first bioactivity for achillin (**3**) described it as a strong antifeedant agent against two grasshopper species [282]. This study showed that a concentration of only 0.5% (% dry weight) of compound **3** was enough to repell *Melanoplus sanguinipes* from feeding. This antifeedant effect was measured qualitatively rather than quantitatively, so it is difficult to accurately assess the full extent of the antifeedant bioactivity. Nevertheless, the rather limited preliminary study [282] was enough to show there was potential application to achillin (**3**). Subsequent in vitro assays followed, showing it had anti-allergic effect (IC_50_ = 100 µM) [279]; increasing chemosensitivity to paclitaxel, with potency comparable to known therapeutics at 100 µM concentration [283]; and antitumor against endocervical cell lines (IC_50_ = 160.3 μg/mL after 72 h [48].

Achillin (**3**) has proven to possess interesting and potent bioactivities tested in vivo. Firstly, the work of Rivero-Cruz et al. [278] showed compound **3** had potent antinociceptive effect in mice assessed using the formalin test. This work started by focusing on the effects of *A. ludoviciana* (edible species), showing it had strong in vivo analgesic and anti-inflammatory effects. Subsequent analysis by HPLC attributed the bioactivity to the presence of two sesquiterpene lactones being one of them achillin and the other dehydroleucodin. Regarding achillin (**3**), it was individually tested using the formalin assay and showed significant activity causing near 50% reduction in formalin wound time with a dose of 17.7 mg/kg (*w*/*w*). Researchers did not specify a mode of action for this activity but cited a previous work [284] attributing the effect to the NF-κB inhibition by sesquiterpene lactones. Another point of critique to the work of Rivero-Cruz et al. [278] would be the heavy emphasis on assaying the plant extract as opposed to the isolated compounds. More extensive testing with the purified compounds would be desirable, producing far more relevant and compelling results. Nevertheless, this work proved achillin possesses interesting antinociceptive bioactivity, which should be studied further, particularly in finding out its mode of action.

Another highly relevant study with achillin (**3**) in animal model was its effect as an inhibitor of meiosis in toad (*Rhinella arenarum*) oocytes was carried out by Zapata-Martínez et al. [285], where denuded toad oocytes were exposed to different concentrations of achillin prior to the necessary hormonal stimulus necessary for meiotic resumption. Results showed a marked overall decrease of germinal vesicle breakdown (GVBD) in occytes with exposure to **3**. It is also worth noting that the response was dose-dependent; a 6 µM concentration of achillin (**3**) showed a ~10% reduction of GVBD compared the control, whereas a 36 µM concentration reduced GVBD by ~35% more than control oocytes. So, it appears achillin (**3**) has meiosis inhibiting potential, which the authors [285] attribute to the covalent bonds formed between achillin’s partially electrophilic center and the nucleophilic center of target molecules. The meiotic inhibitors have been shown to improve human embryonic development in in-vitro fertilization procedures by allowing the embryo to have enough time to finish cytoplasmic maturation [286].

Finally, we present a very recent 2020 paper by Arias-Durán et al. [287] showing achillin’s potential as a smooth muscle cell relaxant. The authors used an ex vivo rat trachea model, where the relaxing effects of increasing concentrations of achillin (**3**) were measured in the rat trachea rings. The results showed achillin (**3**) exhibited almost identical activity to theophylline, a widely used medicine for asthma and chronic obstructive pulmonary disease. The effect seems to be mainly due to a release of nitric oxide and calcium channel blockade influx into the smooth muscle cells of the tracheal rings [286]. This result indicates the great potential as a smooth cell muscle relaxant, with possible applications in the treatment of asthma, bronchospasm and chronic bronchitis.

In conclusion, achillin (**3**) exhibits very interesting bioactivities, mainly anti-inflammatory, meiotic inhibitor, and tracheal relaxant. It is also worth noting that this compound has also been identified as the possible bioactive agent behind interesting *Artemisia* extracts bioactivities, such as allelopathic [276] and anticarcinogenic [275]. It would be very interesting to follow-up on these results with more assays involving the purified compound, although such would possibly require biosynthetic methods, since it is reportedly very difficult to obtain large quantities of purified achillin (**3**) [275]. Nevertheless, there is great potential for its future drug development, but further research is needed.

### 6.4. Tehranolide

Tehranolide (C_15_H_22_O_6_) (**4**) (Figure 1), also sometimes referred to as artediffusin, is a sesquiterpene lactone first isolated from the aerial parts of *Artemisia diffusa* Krasch. ex Poljakov (edible) [288]. Currently, based on The Plant List database, *A. diffusa* name is a synonym of *Seriphidium diffusum* (Krasch. ex Poljak.) Y.R. Ling.

A biosynthesis pathway has been suggested by [289], involving the oxidative cleavage of the Δ^4^ bond of a eudesmanolide derivative, followed by an internal aldol condensation, rearrangement by hydroxy addition and ending with acetal formation. Chemical synthesis has not been described for compound (**4**) which explains why work done with this compound is predominantly centered in a certain few labs and research groups geographically close to the relatively narrow *A. diffusa* natural distribution. There is continued interest in the eudesmanolide chemical synthesis and its derivatives [290,291].

Structurally, tehranolide (**4**) exhibits great similarity to artemisinin (**1**), including the presence of an endoperoxide (C-O-O-C) bridge common to both compounds. This is very noteworthy, since the endoperoxide group is reported to be vital to artemisinin anti-malarial activity [202]. This established connection between structure and function lead to the hypothesis that compound **4** could have antimalarial activity, which was investigated by Rustaiyan et al. [292,293,294], with in vivo results using the purified compound. In this work, the authors use NMRI infected with *Plasmodium berghei*, a widely used malaria animal model organism. The mice were injected daily with doses ranging from 1.7 g to 17 mg (total dose) of HPLC purified tehranolide (**4**). Results showed the lowest dose (17 mg) significantly reduced *P. berghei* parasitemia by ~10% (compared to negative control) 2 days after infection while the highest dose (1.7 g) significantly reduced it by ~14%. It is important to note that, even though dosages varied by a factor of 100, differences between doses were relatively small, and became almost imperceptible 10 days after infection (while still maintaining ~10% difference to negative control after 10 days). We can then conclude that although some dose-dependence effect was exhibited, it was relatively small, and that over time all doses showed almost identical effect when compared between each other.

Some criticism can be made to this work, particularly the fact that total doses were uniformly used instead of doses adjusted to mouse weight. This means that mouse weight fluctuations translated into differing compound systemic concentrations (i.e., “lighter” mice will exhibit higher compound concentrations than “heavier” mice, which could result in exacerbated effect). This ended up not being very relevant, since the results showed relatively small difference between doses, but could have definitely cast doubt upon the results if compound dose-dependency was more exuberant and should be avoided in future assays. Other points open to criticism could be the use of a relatively small sample size (*n* = 5, in triplicate), and the lack of usage of a positive control, in order to compare compound **4′**s efficacy against known used therapeutics. Nevertheless, the work serves to prove that the in vivo anti-malarial bioactivity.

The mode of action for this compound has not been specifically described but, it is thought to be very similar to artemisinin’s (**1**), due to the similar structure [288]. Mechanistically, the antimalarial bioactivity results from the presence of haem or Fe^2+^ resulting from the *P. falciparum* hemolysis. The Fe^2+^ functions as a catalyst to the opening of the peroxide bridge of the compound, which leads to the formation of free radicals, alkylating *P. falciparum* proteins and eventually causing parasite death.

The bioactivity presented above exhibited by tehranolide (**4**) is very interesting and relevant to current society when consider the ever-increasing *Plasmodium falciparum* antimalarial drug resistance [295]. There is indeed an urgent need for novel antimalarial compounds which exhibit potential as a possible therapeutic and the compound **4** was considered a very promise antimalaria agent [296]. Given these facts, it would be expected to see more work done with this compound, hopefully aiming at pre-clinical testing.

Tehranolide (**4**) has also proven to be a very promising anticancer agent, with several recent publications supporting in vivo effect. Much like before, the initial hypothesis was derived from the structural similarity between compound **4** and artemisinin (**1**), whose anticancer activity is discussed above. The earliest account of **4** anticancer bioactivity was described by Noori et al. [297,298]. These papers are arguably some of the most important works on this subject, due to the in vivo nature of the assays and the immunomodulatory insights provided. In summary, a total dose of 5.64 µg of tehranolide (**4**) per mouse was injected intratumor in Balb/c breast cancer mouse models. Results showed this dose significantly inhibited tumor growth by ~75% compared to negative control. Treated animals also exhibited ~2.5× increase in lymphocyte proliferation, as well as ~12% decrease in CD4^+^CD25^+^Foxp3^+^ regulatory T cells, an important factor in tumor tolerance [299], when compared with negative control. These results showed compound **4** had potent in vivo anticancer activity and great potential as an immunotherapeutic regulator. Subsequent follow-up work by the same research group [300] with similarly mice treated (same dose and conditions) revealed a ~80% increase in apoptosis index of tumor cells compared to untreated mice. This result confirms compound **4** indeed possesses in vivo antitumor activity by apoptosis induction (attributed to a tumor cell selective G0/G1 cycle arrest. Finally, Noori et al. [301] describe in detail the mechanism behind compound **4** anticancer activity as being linked to calmodulin and phosphodiesterase type 1 inhibition, as well as cAMP-dependent protein kinase A activity. These processes allow tehranolide (**4**) to selectively inhibit tumor proliferation and induce tumor cell apoptosis.

In conclusion, compound **4** has proven to have great potential as an antimalarial but mainly as anticancer therapeutic. Both of these bioactivities are extremely relevant and important in the modern medical/scientific paradigm, because of the need for novel antimalarials as previously mentioned, as well as rising cancer rates [302]. We hope to see tehranolide (**4**) progress into pre-clinical stages of research with regards to both of these activities.

## 7. Hotpoint Research: *Artemisia* Species and Its Constituents as Strategy to Treat COVID-19 Infection

Caused by a member of the Coronavirus family (CoV), SARS-CoV-2 (COVID-19) has recently posed a potential threat to the survival of human beings on Earth and was declared a global health emergency by the WHO [303]. The therapeutic strategy to treat infection by this coronavirus has used the knowledge and experience acquired in the previous epidemics caused by SARS-CoV-1 and MERS-CoV. To date, there are no vaccines or specific antiviral agents against coronavirus infections, so it is a great challenge for scientists to find treatments for them. Repositioning of drugs already in clinical use is being studied, as a quick response to provide effective treatments in humans and assess other compounds that may be effective against the virus [304]. The WHO proposed *A. annua* as a possible treatment to be considered for COVID-19 treatment, however its efficacy and side-effects must be determined. Additionally, *A. annua* is one of the of Jinhua Qinggan granule ingredients, one of the Traditional Chinese Patent Medicines recommended in 13 therapeutic regimens of COVID-19 in China [305]. The *A. argyi* it was also mentioned as one of the plants that can be used by aromatherapy method of Traditional Chinese Medicine with effects of contagion prevention [306]. Scientific evidence supporting this proposal is partly based on bioactive compounds present in the plant with antiviral effects against hepatitis B, bovine viral diarrhea, and Epstein–Barr virus [307]. It also contains compounds with antioxidant, anti-inflammatory and immunomodulatory properties [176], which would play an important role in controlling the acute inflammatory process triggered by Covid-19 infection. Another research line requiring further attention in the context of acute Covid-19 is the efficacy of the artesunate to ameliorate bleomycin-induced pulmonary fibrosis pathology in rats, possibly by inhibiting profibrotic molecules [308]. Other promising pointers are that *A. annua* showed significant activity in vitro against SARS-CoV-1-2002 (IC_50_ = 34.5 ± 2.6 μg/mL) [309,310]. Tea infusions of *A. annua* and *A. afra* were found to be highly active against HIV virus, although the role of artemisinin is rather limited [311], as were various *Artemisia* species against Herpes simplex virus type 1 [312] and *A. capillaris* against Hepatitis B [169]. In a recent preliminary in silico study, Rolta et al. [313] evaluated the possibility of binding artemisinin (among other compounds) to the cellular ACE-2 receptors via the spicules of the SARS-CoV-2 membrane, as well as ADMET prediction and toxicity analysis. The results obtained support the possibility that artemisinin can act as an antiviral by means of a predictable binding to the receptor, also being non-carcinogenic, non-cytotoxic and safe to administer.

Clearly, greater scientific attention is placed and needed toward *A. annua* and its bioactive compound/derivatives in addressing the treatment of COVID-19 and they need to be further assessed in clinical trials. Kapepula et al. recently published a review drawing attention to the path to be followed and the errors to avoid [314]. And this assessment already started. The phase II clinical study is currently underway (ClinicalTrials.gov identifier: NCT04530617, recruitment), aiming to evaluate the efficacy of *A. annua* and camostat to inhibit viral entry or replication of SARS-CoV-2 virus and their toxicity, administered immediately after COVID-19 positive testing, in mild to moderate disease patients and with high-risk factors such as diabetes, hypertension and obesity, among others.

## 8. Conclusions

This review starts to present an overview of *Artemisia* species traditional use as food, spices, condiment and beverage. The plants are mainly used in salads and tea, as well as to flavoring food and beverages. The leaves are the most used edible part, but the aerial parts, mentioned as “herb”, is also widely used. The nutritional value of *Artemisia* species is also presented and discussed, based on the fatty acid, proteins, sugars, minerals, and vitamin contents reported in the literature.

Studies already published show that the use of *Artemisia* plants is not risk-free. The allergic reactions, mainly allergic rhinitis caused by pollens and skin dermatitis caused by the presence of sesquiterpene lactones, are the most frequently reported and studied adverse effects. Absinthe drinks are reported as causing some adverse side-effects, so its content is legislated. Some *Artemisia* species cause reduction of fertility, so its use is not recommended during pregnancy.

The evaluation, based on clinical studies, of the *Artemisia* formulations effectiveness remains a hot topic. The application of integral plants or extracts as anti-inflammatory agents is deepened and the spectrum of applications broadened to, for example, the preventive effect on hepatitis B cirrhosis, treatment of malaria, anti-allergenic and glycemic control.

Concerning the *Artemisia* constituents, clinical and in vivo studies involving artemisinin and its derivatives show them as efficient antimalarial and anticancer agents. Additionally, the additive or synergistic interactions of artemisinin and derivatives in combination with a wide array of clinically established drugs to combat different cancer are highlighted. The high therapeutic potential is evident in the WHO proposal to investigate artemisinin and derivatives as well as *A. annua* to the treatment of Covid-19 infection. In addition to artemisinin and its derivatives, other sesquiterpene lactones isolated from different species of *Artemisia*, such as santonin, achillin and tehranolide, have been the target of further studies with a view to the development of new derivatives and their application as medicines. These compounds exhibit very interesting activities, in in vivo models, such as immunosuppressant and anti-inflammatory and potent antinociceptive effect. Achillin acts as a meiotic inhibitor and smooth muscle cell relaxant, properties very relevant to improve human embryonic development in-vitro fertilization procedures and to treat asthma and chronic obstructive pulmonary disease, respectively.

Nevertheless, although this review shows the great potential for *Artemisia* species as dietary supplements, functional foods, source of new and more efficient and safe drugs, further research on action mechanism and involving clinical trials on toxicity, adverse side-effects, efficacy and health care uses continue to be needed.

## Figures and Tables

**Table 1:** Application of *Artemisia* species as food, spices, condiments and beverages.

Species	Common Name	Distribution *	Edible Part	Use	Ref.
*Artemisia abrotanum* L.	Southernwood	S. Europe	Young shoots	Flavoring cakes, salads and vinegars; herb tea	[60,61,62,63,64,65]
*A. absinthium*	Mugwort, common wormwood, absinthe	Europe, Asia	Herb	Spice; flavoring beer, wine, vermouth, absinthe, liquors and aperitifs; pelinkovac	[60,61,62,65,66,67,68,69,70,71,72]
*A. afra*	African wormwood	Africa	Herb	Flavoring; preparation of vermouth; as a tea	[60,61,62,63,64,65,66,67]
*Artemisia alba* Turra (syn. *A. camphorata* Vill.)	Camphor absinthe	S. Europe, C. Europe, N.W. Africa	Herb	Spice and flavoring	[60]
*A. annua*	Qing Hao, Sweet sagewort	S.E. Europe to W. Asia.	Leaves	Essential oil in the leaves is used as a flavoring in spirits such as vermouth; as a vegetable	[67]
*Artemisia arborescens* (Vaill.) L.	Silver wormwood	N. Africa, S. Europe	Herb	Spice added to the green tea prepared by Moroccans	[60,65,67]
*Artemisia argyi* H. Lév. & Vaniot	Aicao, Gaiyou, Seomae mugwort	N. Asia, N. Europe, N. America	Leaves, buds, herb	As a tea or other forms of food supplements; dried leaves as a flavoring and colorant for the Chinese dish Qingtua	[46,73]
*Artemisia balchanorum* Krasch.		Turkmenia	Herb	Spice; potherb	[60]
*Artemisia capillaris* Thunb.	Yin Chen Hao	E. Asia—China, Japan, Korea	Leaves, stems, shoots	Soaked and boiled eaten as food supplements in times of famine	[67,74]
*Artemisia carvifolia* Buch.-Ham. ex Roxb.		E. Asia—China, Japan, Himalayas	Leaves	Flavoring for tea and coffee; Young plants—cooked in the spring	[67]
*Artemisia dracunculoides* Pursh.	Russian Tarragon, Tarragon, French Tarragon	N. America. N. Europe. N. Asia—Siberia	Leaves, seeds	Leaves—raw in salads; The N. American Indians bake the leaves between hot stones and then eat them with salt water; Seed—raw or cooked as an oily texture.	[62,67,68,75]
*A. dracunculus*	Tarragon, French Tarragon	S. Europe to W. Asia.	Leaves, young shoots	Leaves—raw or used as a flavoring in soups, oily foods, salads, vinegar, etc.; The young shoots can also be cooked and used as a potherb	[60,62,63,66,67,70,76]
*Artemisia frigida* Willd.	Fringed Wormwood, Prairie sagewort	N. America, N. Asia.	Leaves	The leaves are used by the Hopi Indians as a flavoring for sweet corn	[68,76]
*A. genipi*.	Genepi, black wormwart, black wormwood, génépi noir	S. Europe	Leaves, flower heads	Spice, flavoring for liqueurs	[10,60,61,66,67]
*Artemisia glacialis* L.	Glacier wormwood	C. Europe	Herb, flower heads	Flavoring in vermouth and liqueurs	[10,60,61,67]
*Artemisia granatensis* Boiss.		Spain	Herb	Herb tea	[77]
*Artemisia herba-alba* Asso		Africa, Mediterranean area	Herb	Herb tea; Flavoring tea and coffee	[78]
*Artemisia indica* Willd.		E. Asia—China, Japan, India.	Leaves	Young leaves—cooked and eaten with barley; the leaves pounded with steamed rice dumplings to give a flavor and coloring	[60,70]
*Artemisia japonica* Thunb.		E. Asia—China, Japan, Korea.	Young leaves	Raw as a vegetable or cooked	[70]
*Artemisia keiskeana* Miq.		E. Asia—China, Japan, Korea, E. Russia.	Leaves, shoot tips	Cooked	[67]
*Artemisia ludoviciana* Nutt.	White Sage, Louisiana Sage, Prairie Sage, Western Mugwort	N. America	Leaves, flowering heads	Flavoring or garnish for sauces, gravies, etc.; Used like absinthe; herb tea	[60,61,67,75,76]
*Artemisia maritima* L.	Sea Wormwood	Europe, E. Asia, C. Asia.	Leaves	Spice; flavoring in some Danish schnapps, beer and liqueurs	[60,61,67]
*Artemisia montana* (Nakai.) Pamp.		E. Asia—China, Japan.	Leaves	Young leaves—cooked; herb tea	[79]
*Artemisia pallens* Wall. ex DC.	Davana	N.E. India, Thailand	Herb	Spice; flavoring for cakes, pastries, candy, chewing gum, ice cream, beverages, tobacco; for production of essential oil (davana oil)	[60,61,67]
*Artemisia pontica* L.	Roman wormwood; Small absinthe	S.E. Europe to Siberia, C. Asia	Leaves, herb	Spice, flavoring, like *A. absinthium*	[60,61,66,67]
*A. princeps*	Mugwort mochi, Yomogi	E. Asia—China, Japan, Korea.	Leaves, young seedlings	Raw or cooked in salads and soups; for flavoring and coloring of rice dumplings (‘mochi’)	[60,67,80]
*Artemisia schmidtiana* Maxim.	Sagebrush, Silvermound, Wormwood, Mugwort	E. Asia—Japan.	Stems	Cooked; for flavoring and coloring of rice dumplings (‘mochi’)	[78,80]
*Artemisia sphaerocephala* Krasch.		China	Seed	Seed powder added to noodles and other traditional Chinese foods to improve sensory qualities such as elasticity and chewing quality	[81]
*Artemisia tilesii* Ledeb.	Wormwood, Tilesius’ wormwood	E. Asia, N.W. America.	Leaves, shoots	The fresh shoots are peeled and eaten, usually with oil; Flavoring rice dumplings	[67]
*Artemisia tridentata* Nutt.	Sage Brush, Big sagebrush, Bonneville big sagebrush	N. America	Leaves, seeds	Leaves—cooked, as a condiment and to make a tea with sage-like flavor; Seed—can be roasted then ground into a powder and mixed with water or eaten raw	[61,75,76]
*A. umbelliformis* (syn. *A. mutellina* Vill.)	Alpine Wormwood	Europe—Alps, N. Apennines	Herb, leaves, flower heads	As a condiment; preparation of a tea and a liqueur, often with the addition of absinthe	[10,60,66,67,82]
*Artemisia vallesiaca* All.	Alpine Wormwood, Valais wormwood	Europe—N. Italy, Switzerland, S. E. France	Herb	Flavoring for liqueurs; product of santonin	[10,60,66]
*A. vulgaris*	Mugwort, Common wormwood, Felon Herb, Chrysanthemum Weed, Wild Wormwood	Temperate regions of Europe and Asia	Leaves, young shoots, flowering tops	Flavoring fatty foods; to give color and flavor to rice dumplings (‘mochi’); as a potherb; flavoring in beer and liqueurs	[60,61,66,67,70,71,80]
*Artemisia wrightii* A. Gray.		N. America	Leaves, seeds	Raw or cooked—an oily texture; Seed—ground with water, made into balls and steamed	[75]

* S.—south; E.—east; C.—central; N.—north; W—west; S. E.—south-east; N.W.—north-west; N. E.—north-east.

**Table 2:** Nutritional composition of some edible *Artemisia* species.

Plant Species	Plant Part	Nutrient Composition *	Ref.
*A. absinthium*	Oil cake **	Sugars (9.4%)	[90]
*A. annua*	Leaves	Protein (27.1%); TAA (27.6%), EAA (16.1%), NEAA (11.5%); Crude fat (8.34%); Minerals: K (26.3 mg/g DM), Ca (11.5 mg/g DM), Mg (7.1 mg/g DM), P (7.1 mg/g DM), S (3.9 mg/g DM), Fe (0.2 mg/g DM), Mn (0.2 mg/g DM), Zn (0.06 mg/g DM); Vitamin A (<0.3 μg/100 g DM); Vitamin E (22.63 mg/kg)	[91]
	Inflorescence	Protein (18.4%); Crude fat (10.5%); TAA (18.3%), EAA (10.14%), NEAA (8.11%); Minerals: K (24.6 mg/g DM), Ca (4.4 mg/g DM), Mg (2.3 mg/g DM), P (3.4 mg/g DM), S (4.6 mg/g DM), Fe (0.2 mg/g DM), Mn (0.3 mg/g DM), Zn (0.06 mg/g DM); Vitamin A (<0.3 μg/100 g DM); Vitamin E (19.38 mg/kg)	
	Stems	Protein (10.7%); Crude fat (2.60%); TAA (10.3%), EAA (5.91%), NEAA (4.38%); Minerals: K (13.3 mg/g DM), Ca (0.9 mg/g DM), Mg (0.9 mg/g DM), P (0.7 mg/g DM), S (0.5 mg/g DM), Fe (0.7 mg/g DM), Mn (0.02 mg/g DM), Zn (0.08 mg/g DM); Vitamin A (<0.3 μg/100 g DM); Vitamin E (1.19 mg/kg)	
	Roots	Protein (8.23%); Crude fat (2.13%); TAA (8.01%), EAA (4.34%), NEAA (3.66%); Minerals: K (11.1 mg/g DM), Ca (11.5 mg/g DM), Mg (7.1 mg/g DM), P (7.1 mg/g DM), S (3.9 mg/g DM), Fe (0.2 mg/g DM), Mn (0.2 mg/g DM), Zn (0.06 mg/g DM) Vitamin A (<0.3 μg/100 g DM); Vitamin E (1.36 mg/kg)	
	Leaves	Protein (24.37 mg/100 g); Crude fat (6.07%); TFA (4.19 mg/g FW), SFA (22.9%), UFA (77.1%), MUFA (8.4%), PUFA (68.7%) Carbohydrates (8%); Fibre (14.2%); Vitamins: Tocopherol (2.74%)	[92,93]
	Achene	Lipids: SFA (29.21%), UFA (70.87%), MUFA (13.99%), PUFA (56.88%)	[94]
*A. arborescens*	Leaves	Lipids: TFA (3.31 mg/g FW), SFA (47.4%), UFA (52.6%), MUFA (16.3%), PUFA (36.3%)	[92]
*A. argyi*	Leaves	Protein (22.0 mg/g FW); Free amino acids: EAA (3.71 mg/g DW), NEAA (2.42 mg/g DW), FAA (6.13 mg/g DW); Total lipid (24.7 mg/g FW); SFA (40.8%), MUFA (7.1%), PUFA (52.1%); Total carbohydrates (52.3 mg/g FW); Dietary fiber (39.9 mg/g FW); Minerals: K (74.22 mg/100 g FW), Ca (14.74 mg/100 g FW), Mg (36.64 mg/100 g FW), Zn (0.89 mg/100 g FW), Cu (0.13 mg/100 g FW), Mn (0.76 mg/100 g FW), Fe (3.15 mg/100 g FW); Vitamin C (total ascorbic acid) 2.09 mg/g DW	[46,73]
*A. austriaca* Jacq.	Achene	Lipids: SFA (47.43%), UFA (49.02%), MUFA (9.65%), PUFA (39.37%)	[94]
*A. campestris* L.	Leaves	Lipids: TFA (10.22 mg/g FW), SFA (21.0%), UFA (79.0%), MUFA (3.6%), PUFA (75.3%)	[92]
	Aerial	Crude protein (115 mg/g DM)	[95]
*A. camphorata* Vill.	Leaves	Lipids: TFA (14.82 mg/g FW), SFA (37.4%), UFA (62.6%), MUFA (8.3%), PUFA (54.3%)	[92]
*A capilaris*	Leaves	Lipids: TFA (6.01 mg/g FW), SFA (16.0%), UFA (84.0%), MUFA (4.7%), PUFA (79.4%)	[92]
*A. frigida*	Aerial	Crude protein (17.9%); Minerals: K (18.34 mg/g DM), Ca (7.46 mg/g DM),P (2.54 mg/g DM), Mg (2.17 mg/g DM), Cu (1.1 mg/100 g DM), Mn (24 mg/100 g DM), Fe (20.0 mg/100 g DM) Zn (1.9 mg/100 g DM); Na (5 mg/100 g DM)	[96]
*A. glacialis*	Leaves	Lipids: TFA (8.95 mg/g FW), SFA (21.6%), UFA (78.4%), MUFA (6.8%), PUFA (71.6%)	[92]
*A. gmellini* Weber ex Stechm.	Leaves	Lipids: TFA (14.11 mg/g FW), SFA (25.5%), UFA (74.5%), MUFA (4.5%), PUFA (70.0%)	[92]
*A. herba-alba*	Aerial	Crude protein (103.4–153.6 mg/g DM); Crude fibre (407.9 mg/g DM)	[95,97,98]
*A. jacutica* Drobow	Leaves	Lipids: SFA (61.21–68.12%), UFA (31.88–38.79%)	[99]
*A. ludoviciana*	Leaves	Lipids: TFA (14.28 mg/g FW), SFA (19.6%), UFA (80.4%), MUFA (5.3%), PUFA (75.1%)	[92]
*A. macrocephala* Jaq. ex Bess	Leaves	Lipids: UFA (50.80–65.22%), SFA (34.78–49.20%).	[99]
*A. oleandica* (Besser) Krasch	Leaves	Lipids: TFA (9.84 mg/g FW), SFA (17.6%), UFA (82.4%), MUFA (4.7%), PUFA (77.7%)	[92]
*A. princeps*	Leaves	Lipids: TFA (6.49 mg/g FW), SFA (20.2%), UFA (79.8%), MUFA (5.7%), PUFA (74.1%)	[92]
	Leaves	Lipids: SFA (27.5%), MUFA (35.1%), PUFA (37.4%); Free amino acids: EAA (3.19 mg/g DW), NEAA (2.42 mg/g DW), FAA (5.61 mg/g DW); Vitamin C (total ascorbic acid) 1.01 mg/g DW;	[73]
*A. santolinifolia* Turcz. ex Bess	Leaves	Lipids: SFA (51.8–65.02%), PFA (9.74–44.14%), MFA (4.06–30.85%)	[100]
*A. santonicum* L.	Achene	Lipids: SFA (43.70%), UFA (56.33%), MUFA (8.26%), PUFA (48.07%)	[94]
*A. sieberi* Besser	Aerial	Crude protein (55 mg/g DM); Crude fiber (484 mg/g DM); Minerals: K (13.1 mg/g DM), Ca (15.9 mg/g DM),P (2.5 mg/g DM), Mg (1.8 mg/g DM), Cu (1.37 mg/100 g DM), Mn (2.26 mg/100 g DM), Fe (20.0 mg/100 g DM) Zn (21.2 mg/100 g DM)	[101]
*A. sieversiana* Ehrh. ex Willd	Leaves	Lipids: UFA (64.11–73.23%), SFA (26.77–35.89%)	[99]
*A. sphaerocephala*	Seed	Carbohydrate (73%)	[102]
*A. stelleriana* Bess	Leaves	Lipids: TFA (17.78 mg/g FW), SFA (70.2%), UFA (29.8%), MUFA (1.3%), PUFA (28.4%)	[92]
*A. tridentata* subsp. *wyomingensis* Beetle & A.L.Young	Leaves	Crude protein (15.7%)	[103]
*A. vallesiaca*	Leaves	Lipids: TFA (5.27 mg/g FW), SFA (17.1%), UFA (82.9%), MUFA (9.3%), PUFA (73.6%)	[92]
*A. vulgaris*	Leaves	Lipids: TFA (13.32 mg/g FW), SFA (15.2%), UFA (84.8%), MUFA (3.7%), PUFA (81.1%)	[92]

* Free (FAA), essential (EAA) and non-essential (NEAA) amino acids; total (TFA), saturated (SFA), unsaturated (UFA), monounsaturated (MUFA) and polyunsaturated (PUFA) fatty acids; DW—dry weight; FW—fresh weight; DM—dry matter. ** Oil cake remaining after the extraction of essential oil.

## Data Availability

Not applicable.
